# Promotion or suppression? Does internet use by the Chinese public affect physician trust?

**DOI:** 10.3389/fpubh.2026.1929873

**Published:** 2026-09-08

**Authors:** Haifeng Ding, Xinbin Xia, Jiashan Teng

**Affiliations:** 1 School of Humanities and Management, Hunan University of Chinese Medicine, Changsha, China; 2 School of Public Administration and Law, Hunan Agricultural University, Changsha, China

**Keywords:** digital media, internet use, physician trust, medical services, China

## Abstract

**Objective:**

This study aims to explore the causal relationship between internet use and the level of physician trust among Chinese residents, providing rigorous empirical evidence for understanding the changes in doctor-patient relationships in the digital era.

**Methods:**

Using four waves of panel data from the China Family Panel Studies (CFPS) spanning 2016 to 2022, we construct a two-way fixed effects model to control for individual and time heterogeneity. We further employ propensity score matching (PSM) to alleviate sample selection bias, thereby robustly examining the relationship between internet use and residents’ physician trust.

**Results:**

Internet use is significantly associated with a lower level of residents’ trust in physicians, and this conclusion remains consistent across multiple robustness tests. Heterogeneity analysis reveals that physician trust among individuals with poorer health, lower social status, and no medical insurance is more susceptible to the impact of internet information.

**Conclusion:**

When understanding the relationship between the internet and physician trust, one cannot generalize; instead, refined policy design should be implemented by considering individuals’ health status, social position, and institutional participation. Particularly for groups with high information sensitivity and vulnerable trust, efforts should be made to strengthen online information guidance and health education to prevent them from falling into information cocoons and cognitive polarization.

## 1 Introduction

**Figure 1:**
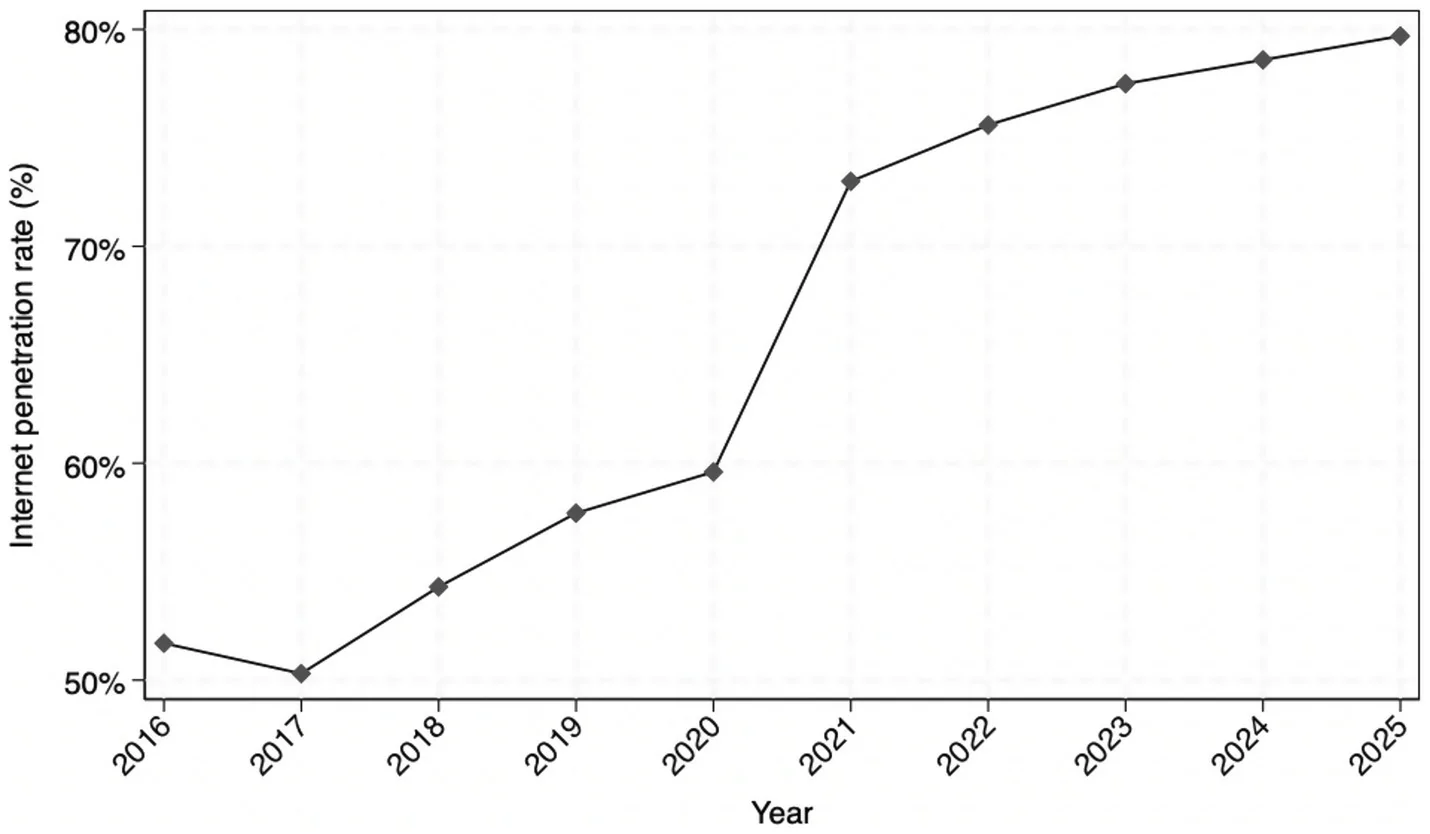
Development trend of internet penetration rate in China (2016–2025).

Physician trust is a cornerstone for building harmonious and stable doctor-patient relationships (1). As an essential component of the coordinated development of medical insurance, medical services, and pharmaceuticals, good physician trust is of great practical significance for maintaining the healthy operation of the medical system and promoting the Chinese-style modernization of the healthcare system. However, in recent years, medical disputes have remained frequent, posing severe challenges to physician trust across society. Meanwhile, digital technologies represented by the internet are reshaping social life with unprecedented breadth and depth, fundamentally transforming information dissemination paradigms (2). According to the 57th Statistical Report on Internet Development in China, as of December 2025, the number of internet users in China reached 1.125 billion, with an internet penetration rate of 80.1%. From 2016 to 2025, China’s internet penetration rate rose from 50% to nearly 80%, showing an overall upward trend (see Figure 1). Similarly, according to a 2022 report, 58.5% of U.S. adults utilized the internet to seek health-related or medical information, with higher incidence among women than among men (3). The continuous rise in internet penetration has made social media, algorithm-recommended news platforms, and short video applications the primary channels for the public to obtain information and form perceptions (4). However, while internet technology empowers the public and promotes information sharing, it also brings a noteworthy derivative effect: the significantly amplified perception of negative information on socially sensitive topics such as doctor-patient relationships.

On the one hand, according to the availability heuristic theory in social psychology, individuals’ judgments of the frequency or probability of events often depend on the accessibility of relevant examples in memory (5). The internet, particularly platforms based on user interaction and algorithmic recommendations, enables extreme and emotional doctor-patient conflict events to spread virally and receive repeated exposure, making them abnormally “available” in public cognition and leading people to overestimate their prevalence. On the other hand, “negativity bias” is a universal human psychological tendency—people pay far more attention to, remember more deeply, and react more emotionally to negative information than to neutral or positive information (6). Reports of medical disputes and accidents that flood cyberspace precisely cater to and continuously activate this cognitive bias. Furthermore, due to internet algorithms, users are prone to the “information cocoon” phenomenon, meaning algorithms continuously push homogeneous content based on user preferences. Individuals with existing doubts about doctor-patient relationships may be trapped in an information environment that reinforces their negative views, leading to increasingly entrenched and polarized cognition (7).

Against this background, there is an urgent need to answer a crucial and insufficiently addressed empirical research question: How exactly does internet development affect residents’ level of physician trust? Although some studies have examined the association between internet use and physician trust, most have focused on correlation analyses using data from a single year or region, lacking rigorous causal analysis based on large-scale national longitudinal data. To address this gap, this study utilizes four waves of nationally representative panel data from the China Family Panel Studies (CFPS) from 2016 to 2022, employing fixed effects models and propensity score matching methods. We aim to scientifically identify the net effect and causal relationship of internet use on physician trust while controlling for individual heterogeneity and selection bias, thereby providing new empirical evidence for formulating physician trust policies in the new era.

## 2 Literature review

Currently, academic research on internet use and physician trust has mainly yielded two perspectives: the “media empowerment theory” and the “media depression theory.”

First is the media empowerment theory, which posits that internet use exerts a significantly positive impact on physician trust. The roots of this argument can be traced back to international scholarship in the early 2000s. Through in-depth interviews with health-information seekers in the United Kingdom, Kivits found that obtaining health information online does not undermine patients’ trust in physicians. Instead, it encourages patients to participate in medical decision making in a more deliberate and proactive manner, thereby improving doctor-patient communication (8). Based on qualitative follow-up research, Sillence et al. reported that patients actively screen online health information. Internet-sourced health materials support individual decision-making and facilitate communication with physicians, while physicians remain patients’ primary source of health advice (9). From the physician perspective, Murray et al. illustrated that the effect of online health information is conditional: accurate online information promotes clinical interaction, whereas inaccurate content may challenge medical authority and damage doctor-patient relationships (10). Li et al. further observed that despite access to abundant online medical resources, patients retain high trust in physicians and still prefer face-to-face consultations. Respondents used online health information rationally for health-related decisions rather than replacing physicians (11). Huang and Tan, based on survey data from residents in four regions across China, constructed a model of how internet health information affects physician trust using structural equation modeling. Their findings indicate that both daily health information and professional diagnosis information directly and positively influence patients’ information perception and promote the establishment of physician trust (12). Li, through empirical analysis of 2021 Chinese Social Survey data, found that public participation in internet-based diagnosis and treatment improves patient trust levels. Additionally, some studies have examined this from the perspectives of government trust and social trust (13). Zhan et al. using 2021 Chinese Social Survey data, empirically found that internet use strengthens differential government trust (14). Wang et al. taking national big data pilot zones as policy representatives of digital technology development, empirically tested how digital technology development reshapes social trust using a difference-in-differences model. Their results show that the implementation of national big data pilot zone policies generally has a positive impact on social trust (15). Nevertheless, Wald et al. pointed out in a comprehensive review that although internet-derived health information facilitates informed patient choices and collaborative doctor-patient decision making, it also brings potential risks such as misinformation and the erosion of medical authority, implying conflicting effects of internet use on doctor-patient relationships (16).

Second is the media depression theory, which argues that internet use exerts a significantly negative impact on physician trust. This perspective is also supported by substantial international literature. Sbaffi and Rowley pointed out that public trust judgments of online health information are shaped by multiple factors such as website design, information sources, and content authority. The ambiguity of these factors may give rise to public concerns over information credibility (17). Daraz et al. offered supply-side evidence via a large-scale meta-narrative systematic review. None of the thousands of assessed health-related websites reached an excellent quality standard, pointing to pervasive quality flaws in publicly accessible online health resources (18). Chen et al. further offered a fresh perspective: trust in online health information itself can impair subjective wellbeing through excessive information-seeking and health-literacy biases (19). Turning to domestic empirical findings, Gao et al. drawing on CFPS 2020 data, found that longer internet usage time significantly lowers public trust in doctors. Although social functions of the internet can partially mitigate such adverse effects, social media still yields an overall trust-reducing effect (20). Hao and Wang, using data from the 2013 Chinese Social Survey (CSS2013), further demonstrated a significant negative correlation between the frequency of online news browsing and patient trust levels (21). Wang and Liu used 2016 China Family Panel Studies (CFPS) data to investigate the relationship between Chinese residents’ internet use behavior and their trust in physicians. The results showed that internet users have significantly lower trust in physicians than non-users (22). Ding and Li, using 2018 CFPS data, examined the impact of mobile internet media on residents’ physician trust, finding that mobile internet media significantly reduces residents’ physician trust levels (23). Li et al. based on Chinese Social Survey (CSS2017) data, found that the public obtaining medical information through online media reduces patient trust (24).

In summary, existing research has conducted extensive empirical tests on internet use and physician trust, yielding rich findings that lay the theoretical foundation and empirical basis for this study’s in-depth analysis. However, upon review, two limitations emerge. On the one hand, existing studies have not reached a unified conclusion on the relationship between internet use and physician trust. On the other hand, existing studies primarily rely on cross sectional data from specific regions to investigate horizontal correlations, lacking longitudinal association analyses derived from multi-year panel data, which to some extent undermines the reliability of research conclusions. Building on this, the present study utilizes four waves of CFPS panel data from 2016 to 2022, employing fixed effects models and propensity score matching methods to rigorously examine the long-term effects of internet use on physician trust, thereby providing novel empirical insights for the formulation of physician trust policies in the contemporary era.

## 3 Research design

### 3.1 Data source

This study employs data from the China Family Panel Studies (CFPS), implemented by the Institute of Social Science Survey at Peking University. The CFPS is a nationally representative long-term longitudinal survey that aims to reflect China’s social, economic, demographic, educational, and health changes by tracking and collecting data at the individual, family, and community levels. The sample covers 25 provinces/municipalities/autonomous regions, with a target sample size of 16,000 households. The survey includes all family members in sample households and encompasses multiple questionnaire modules such as family, adult, and child. To explore longitudinal association relationship between internet use and physician trust, this study primarily selects adult questionnaire data from four survey waves in 2016, 2018, 2020, and 2022 as the analytical sample. During the process of merging the multi-wave data and cleaning, missing values, outliers, and invalid observations were excluded. Ultimately, this study constructs an unbalanced panel dataset comprising a total of 34,996 valid observations. Specifically, the sample distribution across the four waves is as follows: 9,042 in 2016; 10,157 in 2018; 8,393 in 2020; and 7,404 in 2022.

### 3.2 Variable description

Dependent variable. The dependent variable in this study is physician trust. In the four waves of CFPS panel data from 2016 to 2022, there is a question: “How much do you trust doctors?” Respondents are asked to rate their trust on a scale of 0 to 10, where 0 indicates complete distrust and 10 indicates complete trust, with increasing scores representing higher levels of trust.

Core independent variable. The core independent variable in this study is internet use. The CFPS data includes a dedicated mobile phone and internet module for professional measurement. For mobile internet use, there is a question: “Do you use mobile devices such as mobile phones or tablets to access the internet?” For computer internet use, there is a question: “Do you use a computer to access the internet?” This study integrates these two items to generate a new variable for internet use: if respondents use either one, it is coded as 1 (indicating internet use), otherwise as 0.

Control variables. Synthesizing previous research and combining with the research theme, this study selects control variables including age, gender, marital status, and education (25–27). Additionally, considering that physician trust may also be affected by factors such as individuals’ own health levels and social status, this study incorporates health status, social status, and medical insurance participation as control variables into the model (28, 29). Variable definitions and descriptive statistics are presented in Table 1.

**Table 1:** Variable assignment and descriptive statistical analysis.

Variable	Definition	*N*	Mean	SD	Min	Max
Dependent variable						
Physician trust	Continuous variable, 0–10	34,996	6.814	2.336	0	10
Independent variable						
Internet use	1 = Use, 0 = Not use	34,996	0.547	0.498	0	1
Control variables						
Gender	1 = Male, 0 = Female	34,996	0.541	0.498	0	1
Age	Continuous variable	34,996	50.071	14.469	18	95
Marital status	1 = Married, 0 = Unmarried	34,996	0.828	0.377	0	1
Education level	0 = Illiterate/semi-illiterate, 1 = Primary, 2 = Junior high, 3 = Senior high/vocational, 4 = College, 5 = Bachelor, 6 = Master, 7 = PhD	34,996	2.047	1.218	0	7
Household registration	1 = Agricultural, 0 = Non-agricultural	34,996	0.766	0.423	0	1
Health status	1 = Unhealthy, 2 = Fair, 3 = Relatively healthy, 4 = Very healthy, 5 = Extremely healthy	34,996	2.912	1.187	1	5
Social status	Continuous variable, 1–5	34,996	3.015	1.071	1	5
Medical insurance	1 = Participated, 0 = Not participated	34,996	0.919	0.273	0	1

### 3.3 Model construction

Considering that this study is based on four waves of CFPS panel data from 2016 to 2022, and to control for bias in estimation results caused by time-invariant individual heterogeneity and individual-invariant macro time trends, this study employs a two-way fixed effects model for regression analysis. Its basic form is specified as follows:

$$\text{Trus}t_{\mathrm{it}}=\beta_{0}+\beta_{1}\text{Interne}t_{\mathrm{it}}+\gamma Z_{\mathrm{it}}+\alpha_{i}+\lambda_{t}+\varepsilon_{\mathrm{it}}$$ (1)

In Equation 1, Trust_it_ is the dependent variable physician trust, representing the physician trust level of the *i*-th individual in year *t*. Internet_it_ represents the core independent variable internet use, indicating the internet use status of the *i*-th individual in year *t*. *β*_1_ is the estimated coefficient of the core independent variable and also the core parameter of interest in this study. It reflects the net impact of individual internet use on physician trust after controlling for individual fixed effects, time fixed effects, and other observable characteristics. *Z*_it_ represents the control variables. *α_i_* is the individual fixed effect, *λ_t_* is the time fixed effect, *ε*_it_ is the random disturbance term, and *β*_0_ is the constant term of the model.

To alleviate potential self-selection bias in internet use, this study further employs the propensity score matching (PSM) method for robustness testing to estimate the average treatment effect of internet use on physician trust. The core of the PSM method is to construct a counterfactual framework, and its estimated average treatment effect (ATT) formula is as follows:

$$\mathrm{ATT}=E[Y_{\mathrm{1i}}\;|\;D_{i}=1]-E[Y_{\mathrm{0i}}\;|\;D_{i}=1]$$ (2)

In Equation 2, *D_i_* is the treatment variable. In this study, *D_i_* = 1 indicates that individual *i* is an internet user, and *D_i_* = 0 indicates a non-user. *Y*_1i_ is the potential outcome of individual *i* under the “internet use” state, i.e., their physician trust level. *Y*_0i_ is the potential outcome of individual *i* under the “no internet use” state (counterfactual outcome). *E*[*Y*_1i_ | *D_i_* = 1] is the observed mean of physician trust levels for the treatment group (internet users). *E*[*Y*_0i_ | *D_i_* = 1] is the expected value of physician trust levels for treatment group individuals if they did not use the internet.

## 4 Results

### 4.1 Baseline regression

Table 2 reports the baseline regression results regarding the association between internet use and physician trust. Model (1) presents the analysis results without any control variables. The results show that the estimated coefficient of internet use is −0.098 and is significant at the 5% level, indicating that internet use is significantly and negatively associated with residents’ physician trust. Compared to residents who do not use the internet, those who use the internet have lower levels of physician trust. Model (2) adds personal characteristic variables such as gender and age to Model (1). The results show an estimated coefficient of −0.093 with a significance level of 1%, similarly suggesting that internet use is linked to lower physician trust levels. Model (3) incorporates all control variables into the regression model, and the results show an estimated coefficient of −0.099, significant at the 5% level, further confirming the negative correlation between internet use and residents’ physician trust. In summary, Models (1), (2), and (3) consistently indicate that internet use has a significant negative association with residents’ physician trust, i.e., internet use correlates with a decline in residents’ physician trust levels. Specific baseline regression results are presented in Table 2.

**Table 2:** Baseline regression results.

Variable	Model (1)	Model (2)	Model (3)
	Physician trust	Physician trust	Physician trust
Internet use	−0.098^**^ (0.042)	−0.093^***^(0.042)	−0.099^**^(0.041)
Age		−0.101^***^(0.036)	−0.103^***^(0.035)
Marital status		0.036 (0.076)	0.011(0.076)
Education level		0.088^**^(0.040)	0.091^**^(0.040)
Household registration		0.098^**^(0.049)	0.097**(0.049)
Health status			0.069***(0.016)
Social status			0.162***(0.017)
Medical insurance			0.249***(0.053)
Constant	6.868***(0.023)	12.020***(1.854)	11.221***(1.800)
*R* ^2^	0.433	0.433	0.438
Sample size	34,996	34,996	34,996

Data in parentheses are robust standard errors; ^***^, ^**^, and ^*^ indicate significance at the 1, 5, and 10% levels, respectively.

### 4.2 Robustness and Endogeneity tests

#### 4.2.1 Instrumental variable approach

This section adopts the instrumental variable method to address potential endogeneity and simultaneously test the robustness of baseline findings. Drawing on the identification strategy of existing studies (30–32), this paper selects the community average internet usage rate as the instrumental variable for individual internet use. The selection of this instrumental variable is justified by the following theoretical arguments. First, the relevance condition. Infrastructure conditions, digital literacy atmosphere and group demonstration effects within the same community can strongly influence individuals’ internet access behavior. A higher proportion of internet users among other residents in a community makes it more likely for an individual to gain access to the internet through neighborhood learning, information spillover, or shared infrastructure, implying a positive correlation between the two variables. Second, the exogeneity condition. The average internet usage rate of other residents in the community is unlikely to exert a direct impact on an individual’s trust in physicians. Therefore, this instrumental variable is theoretically valid and appropriate.

Table 3 presents the estimation results of the two-stage least squares (2SLS) method. The first-stage regression shows that the coefficient of the instrumental variable (community average internet usage rate) is 0.171 and statistically significant at the 1% level. The first-stage F statistic reaches 389.11, which is far higher than the Stock-Yogo critical value of 16.38 for the 10% threshold, ruling out the problem of weak instrumental variables. Meanwhile, the Kleibergen-Paap rk LM statistic is 343.19 (*p* = 0.000), which further confirms the identifiability of the instrumental variable. The results of the second-stage regression indicate that, after controlling for individual fixed effects and time fixed effects, the estimated coefficient of internet use is −0.535, significant at the 5% level. This result is highly consistent with the baseline regression result in terms of coefficient sign and statistical significance. The core conclusion remains valid after mitigating potential endogeneity bias, which further verifies the strong robustness of the baseline regression results.

**Table 3:** Robustness test results.

Variable	Model (4)	Model (5)
	First-stage regression	Second-stage regression
Community average internet usage rate	0.171***(0.0409)	–
Internet use	–	−0.535**(0.266)
Individual fixed effects	Yes	Yes
Time fixed effects	Yes	Yes
F statistic	389.11	–
Kleibergen-Paap rk LM	343.19 [*p* = 0.000]	–
*R* ^2^	–	0.005
Observations	34,996	34,996

Data in parentheses are robust standard errors; ^***^, ^**^, and ^*^ indicate significance at the 1, 5, and 10% levels, respectively.

#### 4.2.2 PSM for eliminating sample selection Bias

**Figure 2:**
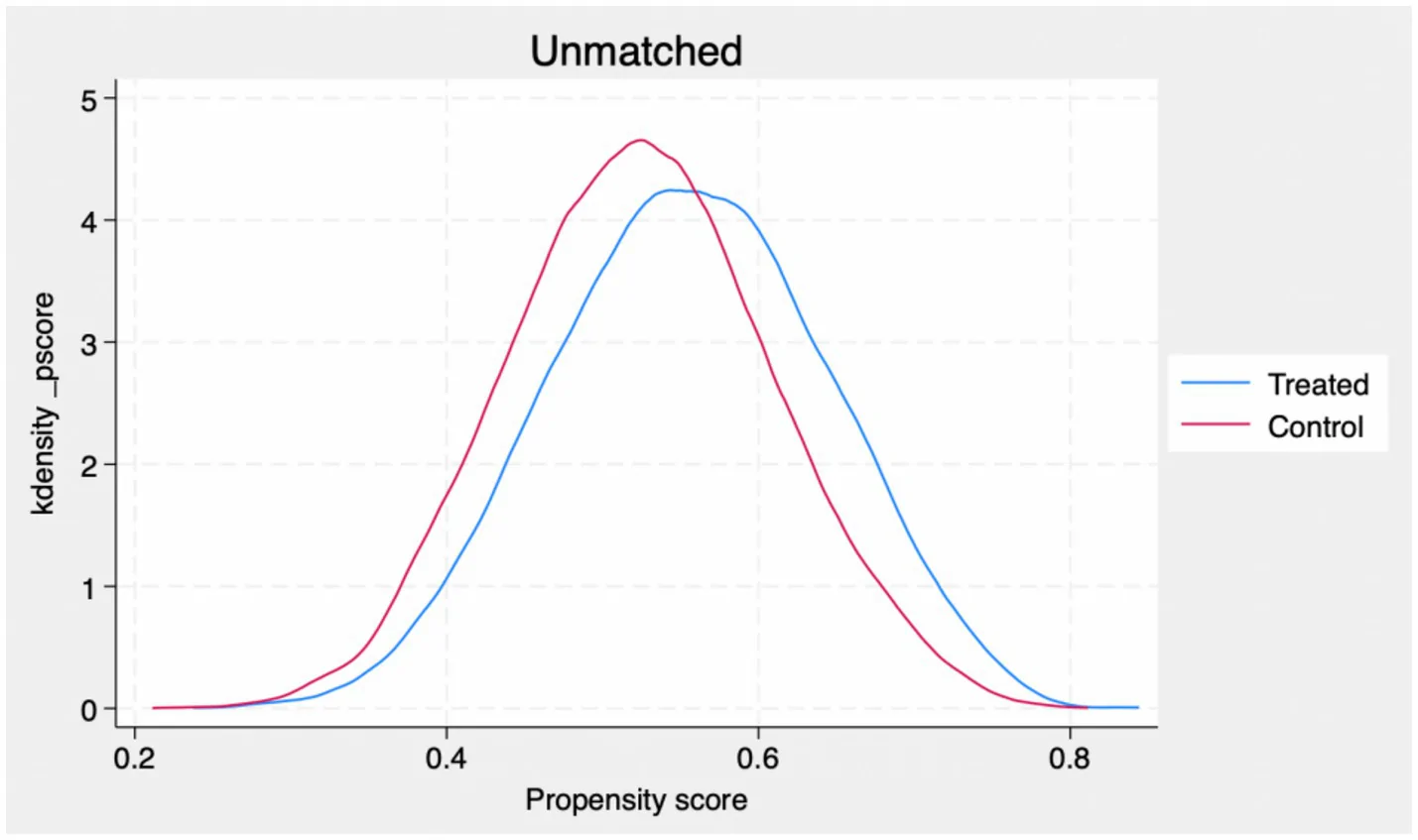
Kernel density function plot before matching.

**Figure 3:**
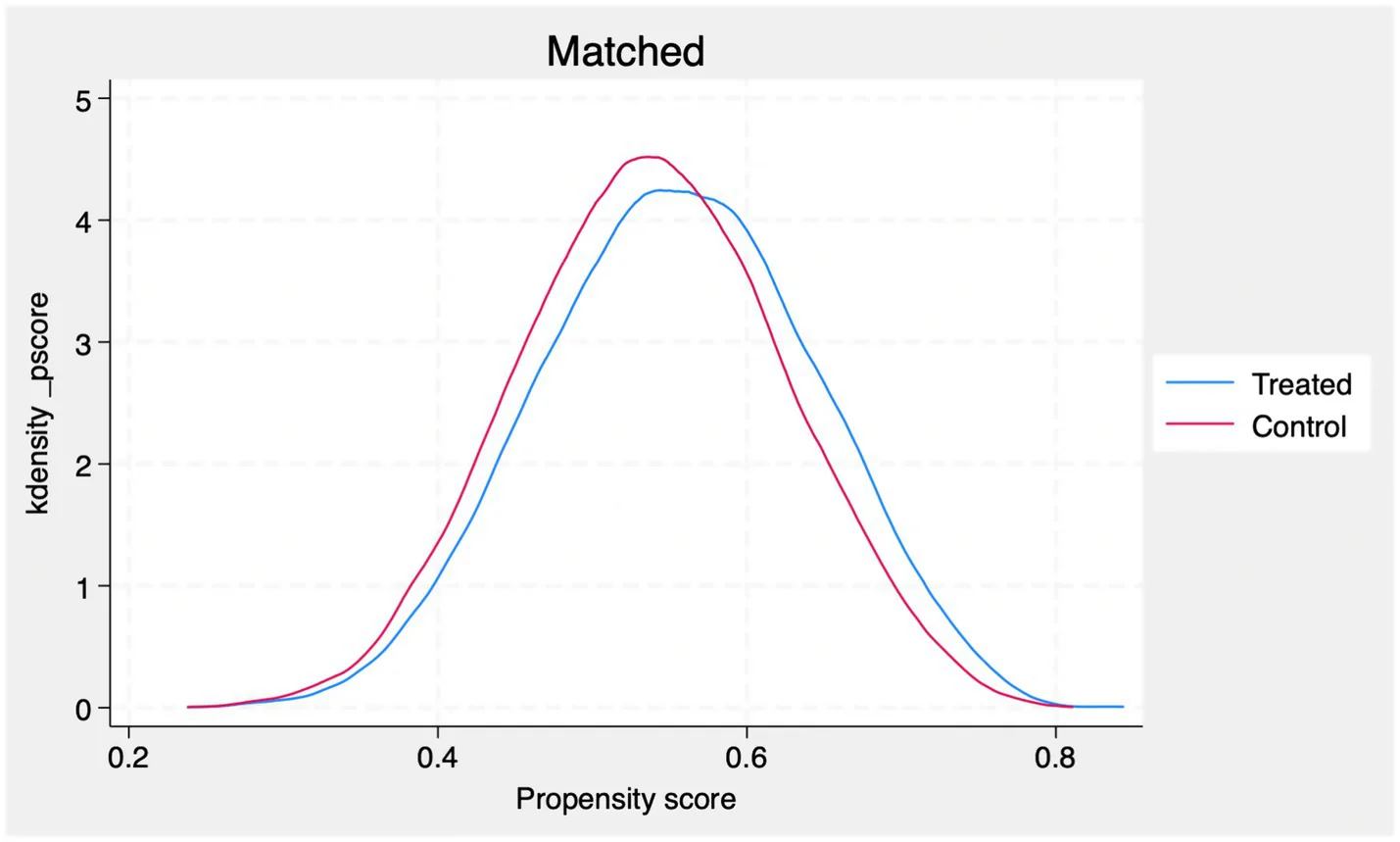
Kernel density function plot after matching.

Since the decision to use the internet is influenced by a series of individual characteristics such as gender, age, and education level, it is essentially a self-selection behavior based on personal endowments and resources, which may lead to endogeneity problems in the model due to sample selection bias. To alleviate the resulting estimation bias, this study employs propensity score matching to construct a counterfactual framework. By matching individuals on observable characteristics, we estimate the average treatment effect on the treated (ATT) to assess the relationship between internet use and physician trust. Referring to existing research, this study simultaneously uses four commonly used methods for estimation: *K*-nearest neighbor matching, *K*-nearest neighbor matching within caliper, radius matching, and kernel matching, to improve the robustness of the results. After matching, this study further conducts balance tests on the samples. Kernel density function plots before and after matching are drawn (see Figures 2, 3). The figures show that the curves of the treatment group and control group overlap more after matching, indicating better matching effects between the treatment group and control group and effective elimination of sample selection bias.

According to the estimation results in Table 4, when using the *K*-nearest neighbor matching method, the average treatment effect (ATT) of internet use on residents’ physician trust is −0.156 before matching and −0.172 after matching. This coefficient captures the difference in scores on the 0–10 physician trust scale and implies that, after accounting for selection bias in observable characteristics, internet use is significantly associated with a 0.172-point lower score in physician trust. The other three matching methods yield similar conclusions: the ATT values corresponding to *K*-nearest neighbor caliper matching, radius matching, and kernel matching are −0.171, −0.197, and −0.196, respectively, meaning internet use corresponds to a lower physician trust score by 0.171, 0.197, and 0.196 scale points correspondingly on the 0–10 measurement scale. The results are highly consistent across different matching methods, indicating that the PSM estimation has good robustness. Additionally, the comparison of effects before and after matching suggests that without effectively controlling for observable sample selection bias, the negative association between internet use and physician trust would be underestimated.

**Table 4:** Propensity score matching estimation results.

Matching strategy	Treatment group	Control group	ATT value	Standard error
Before matching	6.742	6.899	−0.156	0.025
After matching				
*K*-nearest neighbor matching	6.742	6.915	−0.172	0.029
*K*-nearest neighbor caliper matching	6.742	6.914	−0.171	0.028
Radius matching	6.742	6.940	−0.197	0.025
Kernel matching	6.742	6.939	−0.196	0.025

*K*-nearest neighbor matching adopts “one-to-four” matching; the caliper for both *K*-nearest neighbor caliper matching and radius matching is 0.01; both the kernel function and bandwidth in kernel matching use default values.

### 4.3 Heterogeneity test

Constrained by disparities in endowments and resources, the relationship between internet use and physician trust varies significantly across individuals. It is worth noting that prior studies have conducted extensive heterogeneity tests along personal characteristics such as gender, age and educational attainment. To further explore how internet use interacts with residents’ level of physician trust, this paper adopts an interaction-term strategy to examine heterogeneity from three dimensions: individual health status, social status, and medical insurance participation. As shown in Table 5, the association between internet use and residents’ physician trust differs markedly by individual health status, social status, and medical insurance participation.

**Table 5:** Heterogeneity test results.

Variable	Physician trust		
	Model (6)	Model (7)	Model (8)
Internet use × Health status	0.080^***^(0.019)	–	–
Internet use × Social status	–	0.148^***^(0.023)	–
Internet use × Medical insurance participation	–	–	0.261^***^(0.064)
Control variables	Yes	Yes	Yes
Two-way fixed effects	Yes	Yes	Yes
Constant	11.383^***^(1.773)	11.647^***^(1.834)	11.442^***^(1.785)
*R* ^2^	0.438	0.436	0.438
Observations	34,996	34,996	34,996

In terms of health status (Model 6), the negative association between internet use and physician trust weakens significantly as individuals’ health improves, and even turns positive. This suggests that individuals in poorer health are more vulnerable to illness and more sensitive to negative online medical information, rendering their trust more susceptible to such adverse information. In contrast, healthy individuals may acquire valuable health management knowledge via the internet, which is associated with stronger trust in medical providers.

With regard to social status (Model 7), internet use shows a positive link to physician trust for higher-status groups, and this positive correlation grows stronger as social status rises. A plausible explanation is that individuals with higher social status possess stronger information screening capabilities and access to richer medical resources, enabling them to insulate themselves from extreme online anecdotes. By contrast, groups with lower social status have relatively limited information channels and weaker capacity to distinguish information, making them more vulnerable to negative online information.

In respect of medical insurance participation (Model 8), internet use demonstrates a positive correlation for those covered by medical insurance. Individuals enrolled in medical insurance perceive greater merits of the healthcare security system through practical reimbursement experiences. Those lacking medical insurance, however, remain outside institutional protection and face higher medical risks. Negative online narratives concerning difficulties and high costs of accessing medical care as well as doctor-patient disputes can easily trigger psychological anxiety among this group.

## 5 Discussion

### 5.1 Internet use significantly reduces physician trust levels

Based on four waves of CFPS panel data from 2016 to 2022, this study systematically examines the relationship between internet use and residents’ physician trust using a two-way fixed effects model and propensity score matching method. The results indicate that internet use is significantly and negatively correlated with residents’ trust levels in physicians, and this result remains robust after replacing the independent variable, conducting heterogeneity analysis, and eliminating sample selection bias. This aligns with the “media depression theory” perspective that the internet, as a “double-edged sword” of information dissemination, while improving information acquisition efficiency, also exacerbates the public’s negative perception of doctor-patient relationships. In traditional doctor-patient relationships, trust is mostly built on face-to-face professional authority, institutional guarantees, and acquaintance social networks. The popularization of the internet, especially the rise of algorithmic recommendations and social media, has restructured the power dynamics of information distribution and cognitive shaping. The robust negative association revealed in this study demonstrates that the internet in today’s society is not merely a neutral “information channel” but an empowering environment that reshapes the public’s cognitive framework. The empirical results of this study also echo the research conclusions of Wang and Liu (22) and Ding and Li (23) based on cross-sectional data, indicating that the negative impact of the internet on physician trust is not an accidental phenomenon but has robustness across time and samples. Notably, this study further validates this relationship using longitudinal panel data, addressing the limitation of previous studies that mostly relied on cross-sectional data.

### 5.2 The impact of internet use on physician trust exhibits significant heterogeneity

The interaction analysis in this study demonstrates that the relationship between internet use and physician trust exhibits significant heterogeneity across three dimensions: health status, social status, and medical insurance participation. Regarding health status, the negative association between internet use and physician trust diminishes and eventually fades as individuals’ health levels improve. Individuals with poorer health, often driven by frequent personal encounters with medical services, harbor more specific and sensitive evaluative criteria for the healthcare system, making them more vulnerable to negative online medical narratives. In contrast, healthier populations primarily utilize the internet as an empowering tool for health knowledge acquisition and are thus less susceptible to such negative interference. In terms of social status, higher-status groups exhibit a stronger positive association with internet use. A plausible explanation is that higher-status individuals possess stronger information discernment capacities and broader access to medical resources. This enables them to effectively filter out negative online information and leverage the internet to enhance their efficiency in seeking medical care. Conversely, lower-status groups, constrained by weaker information screening skills, are more easily swayed by online negativity. Regarding medical insurance participation, internet use is significantly and positively associated with physician trust among insured group. This indicates that the medical insurance system serves a crucial buffering role in sustaining physician trust. Consequently, internet governance and trust restoration strategies must discard the “one-size-fits-all” approach in favor of differentiated interventions tailored to the specific sources of vulnerability across diverse demographic groups. For populations with poorer health, priority should be given to optimizing their online health information environment; for lower-status groups, efforts should focus on enhancing digital media literacy; and for the uninsured, it is imperative to accelerate institutional coverage to construct a robust safety net for trust.

### 5.3 Policy implications

Based on the above findings, this paper proposes the following three policy implications: First, further improve the governance mechanism for online health information by establishing a negative list of misconduct in health science popularization by medical personnel and a routine spot-check system. It is recommended that provincial health authorities take the lead in building authoritative information dissemination platforms and promote algorithm-based recommendation platforms to implement review and intervention mechanisms for medical and health-related content, thereby curbing at the source the erosion of physician trust by distorted information. Second, shift medical institutions from passive response to active guidance by establishing a closed-loop mechanism for collecting, analyzing, and feeding back on online public opinion. Encourage medical institutions to conduct authoritative science popularization through new media matrices, and integrate public opinion guidance capability into the continuing education system for medical personnel, so as to fill cognitive vacuums and resolve information asymmetry through professional information supply. Third, targeted interventions should be implemented for highly susceptible populations, specifically those with poorer health, lower social status, and no medical insurance. Community health education and medical insurance outreach programs should inherently incorporate modules for enhancing media literacy. Simultaneously, by leveraging contracted family physician services to deliver continuous and authoritative health information, policymakers can utilize institutionalized, face-to-face professional communication to counteract the cognitive polarization induced by online narratives. Ultimately, this strategy ensures the precise allocation of limited public resources while maximizing the marginal benefits of trust restoration.

## 6 Conclusion

Based on four waves of panel data from the China Family Panel Studies (CFPS) from 2016 to 2022, this study systematically examines the relationship between internet development and residents’ physician trust by employing a two-way fixed effects model and propensity score matching method. The study demonstrates that internet use is significantly and negatively associated with the level of physician trust among Chinese residents, and this conclusion holds after variable replacement, multi-dimensional robustness tests, and alleviating observable selection bias. Heterogeneity analysis finds that this negative association is more pronounced among vulnerable groups, specifically those with poorer health, lower social status, and no medical insurance, highlighting a distinct digital stratification effect. The policy implication of this study is that we should move beyond emergency handling of individual extreme events toward systematic governance of the digital medical information ecosystem. Future efforts need to be coordinated in algorithm design, platform responsibility, authoritative narrative construction, and public health literacy education to curb the spread of negative information and cultivate a new ecosystem of rational, mutual trust, and healthy doctor-patient relationships. Several limitations of this study should be acknowledged. First, the measurement of internet use behavior remains relatively broad; future research can further distinguish the differential impacts of different usage scenarios such as information acquisition, social interaction, and online diagnosis and treatment. Second, physician trust is measured using a single-item self-rated scale. While typical for large-scale social surveys like CFPS, it may be subject to certain response biases and might not fully capture the multidimensional nature of trust. Future studies could employ validated multi-item scales for a more comprehensive and robust assessment. Finally, the dynamic formation mechanism of physician trust still needs to be deepened through qualitative research and richer longitudinal data.

## Data Availability

The data from the 2016–2022 China Family Panel Studies (CFPS) are publicly available at http://www.isss.pku.edu.cn/cfps/sjzx/gksj/index.htm, and anyone can access them.
